# Sexual health of ethnic minority MSM in Britain (MESH project): design and methods

**DOI:** 10.1186/1471-2458-10-419

**Published:** 2010-07-14

**Authors:** Jonathan Elford, Eamonn McKeown, Rita Doerner, Simon Nelson, Nicola Low, Jane Anderson

**Affiliations:** 1 School of Community and Health Sciences, City University London, 20 Bartholomew Close, London EC1A 7QN, UK; 2 Terrence Higgins Trust, 8-10 West Street, Bristol BS2 0BH, UK; 3 Institute of Social and Preventive Medicine, University of Bern, Finkenhubelweg 11, 3012 Bern, Switzerland; 4 Centre for the Study of Sexual Health and HIV, Homerton University Hospital NHS Foundation Trust, Homerton Row, London, E9 6SR, UK

## Abstract

**Background:**

Men who have sex with men (MSM) remain the group most at risk of acquiring HIV infection in Britain. HIV prevalence appears to vary widely between MSM from different ethnic minority groups in this country for reasons that are not fully understood. The aim of the MESH project was to examine in detail the sexual health of ethnic minority MSM living in Britain.

**Methods/Design:**

The main objectives of the MESH project were to explore among ethnic minority MSM living in Britain: (i) sexual risk behaviour and HIV prevalence; (ii) their experience of stigma and discrimination; (iii) disclosure of sexuality; (iv) use of, and satisfaction with sexual health services; (v) the extent to which sexual health services (for treatment and prevention) are aware of the needs of ethnic minority MSM.

The research was conducted between 2006 and 2008 in four national samples: (i) ethnic minority MSM living in Britain; (ii) a comparison group of white British MSM living in Britain; (iii) NHS sexual health clinic staff in 15 British towns and cities with significant ethnic minority communities and; (iv) sexual health promotion/HIV prevention service providers. We also recruited men from two "key migrant" groups living in Britain: MSM born in Central or Eastern Europe and MSM born in Central or South America.

Internet-based quantitative and qualitative research methods were used. Ethnic minority MSM were recruited through advertisements on websites, in community venues, via informal networks and in sexual health clinics. White and "key migrant" MSM were recruited mostly through Gaydar, one of the most popular dating sites used by gay men in Britain. MSM who agreed to take part completed a questionnaire online. Ethnic minority MSM who completed the online questionnaire were asked if they would be willing to take part in an online qualitative interview using email.

Service providers were identified through the British Association of Sexual Health and HIV (BASHH) and the Terrence Higgins Trust (THT) CHAPS partnerships. Staff who agreed to take part were asked to complete a questionnaire online.

The online survey was completed by 1241 ethnic minority MSM, 416 men born in South and Central America or Central and Eastern Europe, and 13,717 white British MSM; 67 ethnic minority MSM took part in the online qualitative interview. In addition 364 people working in sexual health clinics and 124 health promotion workers from around Britain completed an online questionnaire.

**Discussion:**

The findings from this study will improve our understanding of the sexual health and needs of ethnic minority MSM in Britain.

## Background

### Men who have sex with men

The continuing rise in new diagnoses of sexually transmitted infections (STI) in the British population has been particularly marked for men who have sex with men (MSM). MSM also remain the group at highest risk of acquiring HIV infection in Britain [1,2].

For the British population overall, STI rates are elevated in black and ethnic minority communities [3-5]. The limited data available suggest that in Britain, MSM from some ethnic minority groups may also be at higher risk for STIs and HIV than white MSM although HIV prevalence appears to vary between ethnic groups [6-8].

### Ethnicity

In Britain, a question on ethnicity was first included in the 1991 census [9]. In the most recent census in 2001, each person in the household was asked: What is your ethnic group? Respondents could tick one of the following: White (British, Irish, Other); Black (Caribbean, African, Other); Asian (Indian, Pakistani, Bangladeshi, Other); Chinese or other ethnic group; Mixed (Black Caribbean and white, Black African and white, Asian and white; any other mixed background). The question on ethnicity will be expanded in the next census (2011) to include "Arab". In the census, white British, Irish or other are classified as "white" while all other groups are classified as "ethnic minority". This classification relies on how an individual defines himself or herself and reflects the group they see themselves belonging to. However, it does not necessarily reflect their place of birth. Some people who belong to an ethnic minority may be born in the UK, others may be born elsewhere. According to the 2001 census, the ethnic minority population in the UK was 4.6 million, or 7.9% of the total population [10]. Nearly half the ethnic minority population described themselves as Indian, Pakistani or Bangladeshi and a quarter as Black Caribbean, Black African or Black Other.

### Ethnic minority MSM

Using figures from the 2001 census, together with data from the second National Survey of Sexual Attitudes and Lifestyles [11], it has been estimated that between 10,000 and 30,000 ethnic minority MSM currently live in Britain.

Homosexuality remains stigmatised in many societies; this may be particularly so in ethnic minority communities in Britain [12-14]. Homosexuality is a criminal offence in most sub-Saharan African and Caribbean countries and throughout much of Asia [15]. As a consequence some ethnic minority MSM in Britain may not identify as gay and, if they do, they might not be "out" to their family, friends, neighbours, colleagues or health professionals.

Anecdotal reports suggest that an increasing number of ethnic minority MSM in Britain are in contact with sexual health services - for both treatment and prevention. However, sexual health services in Britain might be insufficiently aware of same sex behaviour among ethnic minority men and the stigma associated with homosexuality in their communities.

Because of their potentially marginal position in relation to the gay as well as ethnic minority communities, important questions arise about the sexual health of ethnic minority MSM in Britain. For example, are ethnic minority MSM at greater risk of STI and HIV than other men? These questions need to be answered in order to formulate interventions and new ways of working with this population.

### MESH project

We undertook research funded initially by the UK Medical Research Council (July 2006-December 2008), with additional funding from City University London (October 2009-October 2010). This research (the MESH project - **Me**n and **S**exual **H**ealth) has been conducted by researchers at City University London working with colleagues at the Terrence Higgins Trust in Bristol, Homerton University Hospital NHS Foundation Trust hospital in London and the University of Bern, Switzerland. The research was carried out in close collaboration with the British Association for Sexual Health and HIV (BASHH), Gaydar, and a number of community groups and non-governmental organizations working with ethnic minority MSM in Britain, including the Black Gay Men's Advisory Group (BGMAG), NAZ Project London (NPL) and the Terrence Higgins Trust (THT) (full list of collaborators in appendix 1).

The core research question was: How do sexual health services in Britain meet, or fail to meet the needs of ethnic minority men who have sex with men (MSM)?

The main aim of the study was to examine the sexual health of ethnic minority MSM living in Britain. To this end, the primary objectives were to:

• examine sexual risk behaviour and HIV prevalence among ethnic minority MSM in Britain

• investigate (i) the stigma associated with homosexuality in ethnic minority communities in Britain, (ii) discrimination experienced by ethnic minority MSM in Britain, and (iii) their impact on disclosure of sexuality

• investigate access to, use of, and satisfaction with sexual health services among ethnic minority MSM in Britain. Both treatment and prevention services were considered

• investigate the extent to which sexual health services are aware of, and respond to the sexual health needs of ethnic minority MSM. Treatment and prevention services were considered separately.

## Methods/Design

These objectives have been explored using both quantitative and qualitative research methods. Using Internet-based survey methods, the research was conducted in four national samples. These were:

• ethnic minority MSM living in Britain plus two "key migrant" groups of MSM

• a comparison group of white British MSM living in Britain

• NHS sexual health clinic staff in 15 British towns and cities with significant ethnic minority communities

• sexual health promotion staff in the same 15 towns and cities.

In all four samples, those who agreed to participate completed an online questionnaire. A sub-sample of ethnic minority MSM who completed the online questionnaire were invited to take part in an online qualitative interview.

The research received Multi-Centre Research Ethics Committee (MREC) approval from South West MREC in January 2007 (ref: 06/MRE06/71)

In describing the design and methods of the MESH project, we focus on the sampling strategy, recruitment, data collection and data analysis.

### Ethnic minority, key migrant and white MSM - online survey

#### Sampling and recruitment

Research among hard-to-reach groups such as MSM, including MSM from ethnic minorities, is usually based on convenience rather than probability samples [16]. For example, behavioural research among MSM in Britain has primarily been conducted among men recruited in bars, clubs, sexual health clinics [17], gay pride events [18], gyms [19,20] or through the Internet [8,21,22]. While probability sampling would undoubtedly provide a more robust foundation for statistical analysis such an approach is prohibitively expensive in most instances [23]. The high cost is compounded by the fact that there is no sampling frame for MSM. Convenience samples have the advantage of being affordable and also provide the opportunity to focus on men with characteristics which may be of particular interest, eg ethnic minority MSM [16]. The disadvantage is that such samples may introduce selection bias. This bias can be partially overcome by recruiting MSM from more than one source [22].

For the MESH project, we recruited a national sample of ethnic minority MSM from a number of different sources both online (through the Internet) and offline (through sexual health clinics, bars, clubs, social networks and the press). We liaised closely with community groups and individuals working with ethnic minority MSM in Britain during this phase of the project (see appendix 1 for list of community representatives involved in the study). As well as recruiting MSM who belonged to an ethnic minority using the census classification (eg Black African, Indian, Chinese, etc) we were advised by the community groups to also include two key groups of migrants in the study; MSM from *South and Central America *and MSM from *Central and Eastern Europe*. Some of the community groups had noted a recent increase in migration of MSM from South and Central America and were providing outreach services for this group of men (for the purposes of this study we included Mexico as a Central American country). With the accession to the European Union of 10 Central and Eastern European countries since 2004, there has also been a sizeable movement of MSM from these countries to the UK (i.e. from Bulgaria, Czech Republic, Estonia, Hungary, Latvia, Lithuania, Poland, Romania, Slovakia and Slovenia).

We recruited our comparison group of white British MSM primarily online. Our earlier research found that advertising on Gaydar attracts several thousand MSM most of whom (> 90%) describe themselves as "white" while the remainder say they are ethnic minority [22,24]. Consequently, online recruitment will generate a substantial number of white MSM for the comparison group as well as attracting men from the "key migrant" groups and from ethnic minority backgrounds. Our earlier research also showed that MSM recruited through the Internet are broadly comparable with MSM in a national probability sample in Britain in terms of education, social class, area of residence, country of birth, alcohol consumption, age at first sex and HIV testing history [25]. However, men recruited through the Internet were on average younger than men in the probability sample and more likely to report high risk sexual behaviour or a history of sexually transmitted infections.

#### Online recruitment

Between August 2007 and April 2008, we promoted the MESH project using banner advertisements on community websites (e.g. http://ukblackout.com, http://gaysia.co.uk, http://imaan.org.uk), health promotion websites (eg THT, GMFA, Health Gay Living Birmingham, LGBT Scotland) and on dating and social networking websites known to be used by ethnic minority MSM (e.g. http://manhunt.net, http://blackgaychat.com). We also promoted the project on Gaydar, the most popular gay dating site in the UK used by white as well as ethnic minority and "key migrant" MSM. See appendix 2 for a complete list of websites used to advertise the online questionnaire.

Clicking on the banner took men to the homepage of the online survey for MSM. This provided information about the study. Men who agreed to complete the questionnaire then did so after providing informed consent - all online. The online survey was developed by a professional web designer and hosted on Gaydar's server.

In January 2008 Gaydar also sent a personal online message to its UK subscribers promoting the MESH project with a direct link to the project's homepage and online questionnaire. This message was sent to all UK subscribers regardless of ethnic background or nationality.

Several community groups (e.g. LYC, Imaan, THT) had email lists of their members whom they contacted on a regular basis. Early in 2008 we sent a short email describing the project to the people who managed the lists and asked them to forward the email to all their list members. The email contained a direct link to the online questionnaire for MSM.

#### Offline recruitment

We used a variety of methods for recruiting ethnic minority MSM "offline" by advertising the project and online questionnaire in sexual health clinics, bars, clubs and the media. Since we did not have sufficient resources to target "offline" every urban and rural area in Britain, we chose the 15 British towns and cities with the largest ethnic minority populations according to the 2001 census [10]. The cities and towns are (in alphabetical order): Birmingham, Bradford, Brighton, Bristol, Cardiff, Glasgow, Leeds, Leicester, Liverpool, London, Luton, Manchester, Newcastle, Nottingham and Sheffield.

We approached individual sexual health clinics in the 15 towns and cities for assistance in recruiting ethnic minority MSM (see the section on "Sexual health clinic staff - online survey" for further details on how we identified the clinics). In October 2007, we sent the participating sexual health clinics posters, postcards and credit-card-size cards advertising the MESH project. We asked the clinics to promote the project among ethnic minority MSM using their services over the next five months (that is, between October 2007 and February 2008). They were asked to display the posters and leave postcards in the waiting areas, and to tell ethnic minority MSM about the project if the opportunity arose during a clinic consultation. The posters and postcards provided the URL address of the MESH project homepage http://www.meshproject.org.uk where men could get information about the study and access the online questionnaire. Men who agreed to complete the questionnaire then did so after providing informed consent - all online.

Forty clinics (22 in London,18 outside London) agreed to recruit ethnic minority MSM for the study (see Additional file 1 for list of participating clinics). We sought Research and Development (R&D) approval from each of the local NHS Trusts for their clinic(s) to participate in the project. Approval was obtained for 38 of the 40 clinics (London, 20; outside London 18).

We maintained regular contact with the clinics during the recruitment period. In January 2008 we sent new posters and postcards, incorporating specific images of Black, South Asian and East Asian men, to all the clinics as well as a short PowerPoint presentation about the project which they could use in their weekly staff meetings.

In each of the 15 towns and cities we also contacted a local HIV prevention or health promotion organisation (described in more detail in the section "Sexual health promotion staff - online survey"). As with the clinics, we sent them posters and postcards in October 2007. We asked their outreach workers to distribute these in gay venues (bars, clubs, drop in centers, etc) in the course of their work over the next five months (October 2007 to February 2008). The postcards provided the URL address of the MESH online questionnaire. Four organisations in London and 15 outside London agreed to promote the MESH project among ethnic minority MSM (see appendix 3 for list of participating organisations). In London, for example, our collaborating partners agreed to distribute promotional material in 81 venues across the capital. We maintained regular contact with the health promotion organisations during the recruitment period. In January 2008 we sent new posters and postcards to all the agencies.

In addition we distributed postcards at two black gay pride events in London in August 2007. We also employed casual workers ourselves who distributed postcards between December 2007 and February 2008 advertising the project in clubs and venues in London which attract a large number of ethnic minority or key migrant MSM. These were Bootylicious (Black Caribbean and African MSM), Exilio (South American MSM) and Club Kali (Indian, Pakistani and Bangladeshi MSM). We distributed cards in Kudos, Heaven and City of Quebec, venues which attract a mixed clientele including men from ethnic minority backgrounds.

We also placed advertisements in the gay press in London (QX magazine, Boyz, Out in the City), Manchester (North West Out) and Newcastle (Out North East) in October, November and December 2007. The principal investigator was interviewed on Gaydar radio towards the end of 2007 and City University London sent out a press release at the same time.

Finally Gaydar sent a Freshers' pack to all university LGBT societies in the UK in September 2007 which included postcards advertising the MESH project.

#### Number of MSM recruited

Over 19,000 people clicked through to the homepage of the MESH online questionnaire and gave their consent to take part in the survey. Of these, 17,425 matched the inclusion criteria, ie they were male, over the age of 18 years, lived in the UK and reported having sex with a man at some time in their life. Of these, 1241 described themselves as ethnic minority (table 1). A further 173 men were migrants from Central and South America and 243 were migrants from Central and Eastern Europe. In addition, 13,717 men said they were white British. Three hundred and ninety-four white Irish and 1595 white "other" men also took part in the survey but they were excluded from the analysis.

**Table 1:** Number of ethnic minority MSM who took part in the online survey

Ethnic group	Number of respondents
Black Caribbean	140
Black African	96
Black other	22
Black Caribbean and white	82
Black African and white	59
Indian	199
Pakistani	91
Bangladeshi	19
IPB* and white	70
Chinese	166
Other Asian	152
Arab	66
Other ethnic group	79
	
All ethnic minority respondents	1241

*IPB: Indian, Pakistani or Bangladeshi

Of the 15,374 men (13717 + 1241 + 173 + 243), 13649 (88.8%) completed the whole questionnaire (white MSM 89.3%, ethnic minority MSM 83.2%, "key migrant" MSM 86.5%). The remaining men dropped out before the end.

Of the white British men, 96% said they had heard about the survey through Gaydar (92%) or another website (4%). By way of comparison, 72% of ethnic minority MSM said they had heard about the survey through Gaydar (65%) or another Internet site (7%). The remaining ethnic minority MSM (28%) had heard about it elsewhere, for example, in a sexual health clinic, at a venue or through friends - this varied between ethnic groups. The overwhelming majority of "key migrant" MSM had also heard about the survey through Gaydar (Central/Eastern European MSM, 89%, Central/South American MSM, 82%) or other websites (6% and 4% respectively).

Estimating a response rate for the MSM recruited online is problematic [26-28]. It is impossible to gauge how many men saw the banners and pop-ups advertising the online survey, read the online message or opened an email promoting the survey. Nor do we know what percentage of those seeing the banners, online message or email went on to complete the questionnaire. Based on estimates provided by Gaydar on the number of people using their Internet chatrooms and profiles during the survey period, it is likely that less than one percent of all their users completed the questionnaire. This level of response is standard for online surveys. Equally, we are not able to estimate a response rate for those recruited offline (eg. in a sexual health clinic or in a commercial venue) since we do not know how many people saw the posters or picked up postcards advertising the project in the different locations.

#### Data collection

The online questionnaire sought detailed information on the men's socio-demographic characteristics (age, ethnicity, country of birth, place of residence, employment, education), sexual orientation and HIV test history (date and result of last test). Questions were also included on access to, use of, and satisfaction with sexual health services including recent history of STIs. Respondents were asked about their sexual risk behaviour. If men reported unprotected anal intercourse (UAI) in the previous three months, we asked about the type of partner (regular or casual), as well as the HIV status and ethnicity of their partner(s). In addition we asked men about their sense of belonging to the gay community and to their "ethnic minority community" and about any discrimination they had experienced because of their sexuality or ethnicity. We also asked whether they had told others about their sexuality and if so whom. Data were collected on potential confounding factors in relation to sexual behaviour such as recreational drug use, alcohol consumption, mental health and attitudes towards new treatments for HIV. In total, the online questionnaire comprised 143 questions. However, most men would have been asked to answer less than this number, since they would have been routed round certain sections depending on their answer to specific questions.

Standardized and validated questionnaire items were used extensively. For example, questions on ethnicity were based on the 2001 census [10] (plus the new category "Arab" which will be included in the 2011 census) and behavioural questions were taken from other social and behavioural research projects [19,22,29]. The questionnaire was piloted both offline and online among gay men at the developmental stage of the study and revised in the light of feedback and comments.

The questionnaire was only in English because of financial constraints. All questionnaires were anonymous and confidential. They contained no information that allowed an individual respondent to be identified. Identifiers such as IP addresses were removed from the online questionnaire before the data were downloaded into a database.

### Ethnic minority MSM - online qualitative interview

The MESH project provided us with an opportunity to adopt an innovative approach to conducting one-to-one qualitative interviews - by conducting them online using email [30-32].

#### Sampling and recruitment

After completing the online questionnaire, ethnic minority MSM were asked if they would be willing to receive information about an in-depth one-to-one interview which would be conducted online. If they were interested in finding out more about the one-to-one interview they were asked to provide an email address so that we could contact them. Before any of the email addresses were forwarded to us, the link between the questionnaire and the email address was broken. Consequently, the questionnaires remained totally anonymous. We did not ask White British MSM or "key migrant" MSM to take part in the online qualitative interview.

Those men who provided an email address were subsequently sent (by email) an information sheet about what the online qualitative interview would entail. They had the opportunity to ask questions by email before providing consent to take part. The researcher (EM) explained that over the next three to four weeks he would exchange emails with the respondent to explore in greater depth some of the topics covered by the online survey. It was anticipated that the email interview would take 2 or 3 hours of the participant's time overall. All communication was via email.

Once a respondent had provided consent by email for a one-to-one qualitative interview, the researcher sent the first set of questions.

#### Number of ethnic minority MSM recruited

327 ethnic minority MSM provided an email address so that they could find out more about the one-to-one interview. EM sent an information sheet by email to all 327 men of whom just over a third (123) provided consent to take part in a one-to-one interview. EM then sent the first set of questions to these 123 men of whom 97 replied. EM sent the second set of questions to each of the 97 men; 67 men replied to this email. Up to 6 "interview-emails" were sent. There was attrition at every stage (table 2).

**Table 2:** Online qualitative interviews with ethnic minority MSM

	Number of men
Provided an email address to find out more about the one to one email interview	327
Agreed to take part in a one-to-one email interview and returned a consent form	123
Responded to the 1st interview-email	97
Responded to the 2nd interview-email	67
Responded to the 3rd interview-email	50
Responded to the 4th interview-email	40
Responded to the 5th interview-email	25
Responded to the 6th interview email	16

#### Data collection

The online qualitative interview consisted of a series of email exchanges between the researcher (EM) and the participant over a period of three to four weeks. Each email contained up to ten questions.

The initial email for the interview included background questions about age, residence, ethnicity, place of birth, education, employment, relationship status and HIV status. Subsequent emails focused on substantive areas such as sexual and cultural identity, disclosure of sexual orientation to family and social networks, experiences of stigma and discrimination, sexual preferences in relation to ethnicity and affiliation with gay community. The email exchange took the form of a semi-structured interview whereby EM's questions in one email were often based on responses submitted by a participant in a previous email.

At the end of the interview the text was copied and pasted into a Word document and given a serial number. The original emails were deleted. Email addresses and serial numbers were stored in a separate password-protected database to which only members of the research team had access. It was impossible to link the emails exchanged as part of the qualitative interview with the online questionnaire the respondent had previously completed.

All those respondents who replied to at least the first two "interview emails" (n = 67) were included in the qualitative analysis. The 30 men who only responded to the first email (97-67 = 30) were not included in the analysis since this email only sought information on social and demographic background characteristics. Subsequent emails were more probing and yielded substantive qualitative material. The length of individual email replies varied considerably.

### Sexual health clinic staff - online survey

#### Sampling and recruitment

One of the objectives of the research was to investigate the extent to which sexual health services are aware of the needs of ethnic minority MSM and how they respond to them. Treatment and prevention services were considered separately. To this end, we wanted to survey doctors, nurses, health advisors, counselors and psychologists working in NHS sexual health clinics in Britain. We only included clinics in the 15 British towns and cities with the largest ethnic minority populations according to the 2001 census (as described above) for two reasons. First, staff in these clinics were more likely to have had some experience of seeing and treating ethnic minority MSM in their clinics than staff in towns with smaller ethnic minority populations. Secondly, since we did not have sufficient resources to include every sexual health clinic in Britain, we focused on those most likely to provide services to ethnic minority MSM patients.

From the British Association of Sexual Health and HIV (BASHH) website http://www.bashh.org we were able to identify all the National Health Service (NHS) sexual health clinics in the 15 target towns and cities in Britain (30 clinics in London, 19 outside London) (Additional file 1). Using this sampling frame of NHS sexual health clinics, we identified a key contact in each clinic and provided them with information about the study by email and telephone. We emphasized that the research was being conducted in collaboration with BASHH. If requested to do so, we visited the clinic to discuss the research in person.

The sexual health clinics were asked to do two things: (i) to promote the project among their ethnic minority MSM clinic attendees (as described in the section "Ethnic minority, key migrant and white MSM - online survey"); (ii) for the clinical staff (doctors, nurses, health advisors, counselors, psychologists) to complete an online questionnaire concerning the needs of ethnic minority MSM using their clinic. Of the 49 clinics identified through the BASHH website, 40 (82%) initially agreed to participate in the research (London 22/30, 73%; outside London 18/19, 95%). The remaining clinics did not respond to our emails asking them to take part in the survey. Most of the London clinics that did not respond to our emails were in outer London.

The 40 clinics which were willing to take part in the research were located in 31 hospital trusts, each with its own Research and Development (R&D) department. In each clinic we identified one member of staff who agreed to be the designated Local Collaborator at his or her clinic for the purpose of receiving R&D approval for the research. We did not need to go to Local Research Ethics Committees since the research had received Multi-centre Research Ethics Committee (MREC) approval (ref: 06/MRE06/71). R&D approval was granted for 38 of the 40 clinics (95%) that had expressed their willingness to take part in the research. For two of the clinics who had said they were willing to take part in the research, R&D approval had not been granted by the time the online questionnaire for staff went live, so they had to be excluded. We required R&D approval not only for the clinical staff to participate in the research but also for the clinic to promote the online survey among their ethnic minority MSM attendees (as described in an earlier section).

Liaising with R&D departments, and completing the necessary paperwork, required at least one day of EM's time for each trust, ie at least 31 days in all. Completing formalities with the R&D offices was equivalent to more than six week's full-time work for the researcher. The average time between making initial contact with the R&D office in the hospital trust and their approving the clinic's participation in the study was six weeks (range 3 - 28 weeks).

Once R&D approval had been obtained, the Local Collaborator in each clinic was asked to identify and enumerate (but not name individually) the staff in their clinic who would be eligible for the questionnaire phase of the study. In November 2007 we sent an email to the key contact in each clinic and asked them to forward the email to all eligible clinical staff. The email briefly described the study and asked each staff member to complete a questionnaire concerning the sexual health needs of ethnic minority MSM. The questionnaire could be accessed online, with a direct link from the email to the questionnaire's homepage. After reading an information page, clinic staff were then asked to provide online consent before proceeding to the questionnaire.

We sent reminder emails in December 2007, in January 2008 and February 2008 to the key contact in each clinic, with a request that they forward a reminder to the staff in their clinic who were eligible to take part in the study. Recruitment stopped in March 2008.

Of the 38 clinics that agreed to take part in the survey and had R&D approval, staff from 36 clinics completed a questionnaire (London 19, outside London 17). Clinics from all but one of the target towns and cities took part in the staff survey.

So, of the 49 NHS sexual health clinics initially identified, staff from 36 clinics (73.5%) completed an online questionnaire (London 19/30, 63.5%; outside London 17/19, 89.5%). Overall 364 clinic staff completed an online questionnaire (London 152, outside London 199, unspecified 13) (table 3).

**Table 3:** NHS sexual health clinics in the 15 target towns and cities

	London		Outside London		Total	
	
	n	%	n	%	n	%
						
Number of NHS sexual health clinics in the target towns and cities	30	100.0	19	100.0	49	100.0
Number of clinics that agreed to take part in the study	22	73.3	18	94.7	40	81.6
Number of clinics where R&D approval was granted	20	66.7	18	94.7	38	77.6
Number of clinics whose staff took part in the online survey	19	63.3	17	89.5	36	73.5
Number of staff who completed a questionnaire in 36 clinics	152	-	199	-	364*	-

Number of staff who were invited to take part in the survey**	493	100.0	498	100.0	991	100.0
Number of staff who completed a questionnaire**	134	27.2	177	35.5	311	31.4

*13 respondents did not indicate which town or city they worked in

**Based on data provided by 30 of the 36 participating clinics

The Local Collaborator in 30 of the 36 clinics provided information on the number of people in their clinic who were sent the email link to the clinic staff questionnaire. Using this information we estimated that 991 clinic staff were eligible for the survey in these 30 clinics (493 in London, 498 outside London). 311 people completed questionnaires in these clinics yielding a response rate of 31.4% (London 27.2%, outside London 35.5%) (table 3).

#### Data collection

The online questionnaire for sexual health clinic staff comprised both closed questions (with tick-box responses) and open questions where respondents could provide answers in their own words. The questionnaire was developed in close partnership with the BASHH Education Committee and was piloted among BASHH members.

The questionnaire sought information on:

• age, sex and ethnicity of the respondent

• training in relation to ethnicity, cultural awareness and sexuality

• experience of providing treatment and care for ethnic minority MSM

• main issues and problems facing ethnic minority MSM

The clinical director of each clinic was asked additional questions about services for MSM and men from ethnic minority backgrounds.

Since the questionnaire contained seven open questions we developed a coding scheme whereby answers to a specific question could be grouped under a number of headings. The coding scheme was initially developed by EM and then further scrutinized and finalized in a series of meetings with JE. We employed an experienced researcher to then code the answers to the open questions using the coding scheme.

### Sexual health promotion staff - online survey

#### Sampling and recruitment

In the previous section we described how we identified and recruited staff in sexual health clinics for the MESH project. Here we describe how we identified and recruited staff working in sexual health promotion and HIV prevention services.

Initially we targeted health promotion staff in the 15 British towns and cities with the largest ethnic minority populations according to the 2001 census (described above). In Britain, the Terrence Higgins Trust (THT) is responsible for delivering a substantial part of the HIV prevention programme for MSM through "CHAPS". CHAPS is a partnership of gay men's health promotion organisations in Britain with a national remit for HIV prevention. Working with the THT and the CHAPS partnership, we identified HIV prevention projects in the 15 target towns and cities in Britain.

Using this sampling frame of sexual health promotion services, we contacted the manager of each project by email or telephone and provided them with information about the study. We emphasized that we were conducting the research with the Terrence Higgins Trust CHAPS partnership along with community groups working with ethnic minority MSM. The sexual health promotion projects were asked to do two things:(i) to promote the project among ethnic minority MSM in their area, through their outreach work (described in the section "Ethnic minority, key migrant and white MSM - online survey" above); (ii) for the health promotion staff to complete an online questionnaire concerning the needs of ethnic minority MSM using their services.

If the sexual health promotion project was part of a hospital or primary care trust we obtained R&D approval as described for the sexual health clinics above. However, the majority of the projects were not part of a hospital or primary care trust and were in a position to provide consent to participate without reference to another body. Since we had Multi-Centre Research Ethics Committee approval we did not need to seek ethics approval from local committees.

We identified a key contact in each sexual health promotion project. In January 2008 we sent an email to the key contact in each project and asked them to forward the email to all their staff and volunteers. The email briefly described the study and asked each staff member or volunteer to complete a questionnaire concerning the sexual health needs of ethnic minority MSM. The questionnaire could be accessed online, with a direct link from the email to the homepage. The sexual health promotion staff had their own homepage, separate from the homepages which the MSM or sexual health clinic staff had accessed. Staff could then complete the questionnaire, once they had provided informed consent, online. We sent reminder emails to the key contacts in February and March 2008.

It soon became clear that recruiting health promotion staff through projects and services in the 15 target towns and cities was problematic. Only 30 health promotion staff or volunteers in the target towns and cities completed the online questionnaire during the first six weeks of recruitment. In addition, most key contacts were unable to provide complete information about the number of staff and volunteers in their team.

The annual CHAPS conference held in Nottingham in March 2008 provided us with an opportunity to modify our recruitment strategy. The CHAPS conference focuses on HIV prevention among MSM in Britain and attracts a large number of sexual health promotion workers from around the country. Over two hundred people attended the conference. After the conference we asked the THT staff who organized it whether they would send, on our behalf, an email to all the delegates inviting them to take part in the health promotion staff survey. The THT agreed to do this. We drafted a brief email describing the study and asked each delegate to complete the questionnaire if they were directly engaged in HIV prevention or sexual health promotion work with MSM in Britain. The THT sent this email to conference delegates in April 2008 with a direct link to the online questionnaire. This approach attracted a further 94 respondents; some, but not all, worked in one of our target towns and cities. Recruitment for this part of the study stopped in May 2008.

Overall, 124 health promotion workers completed an online questionnaire; 80 (65%) worked in one of the 15 target towns and cities while the remainder worked elsewhere in Britain. We were not able to estimate a response rate since we did not have complete information on the denominator, i.e. the total number of staff and volunteers engaged in HIV prevention and sexual health promotion in Britain.

#### Data collection

The questionnaire comprised both closed questions (with tick-box-type responses) and open questions where respondents could provide answers in their own words. The questionnaire was developed in close partnership with the CHAPS partnership and was piloted among health promotion workers before being finalized.

Information was sought from health promotion/HIV prevention service providers

on:

• age, sex and ethnicity of the respondent

• services provided for ethnic minority MSM

• main issues and problems facing ethnic minority MSM

The questionnaire contained five open questions. Since the questions were the same, or similar to those we used in the questionnaire for sexual health clinic staff we used the same coding scheme to group answers to a specific question under a number of headings. We employed an experienced researcher to code the answers to the five open questions for the 124 respondents.

### Data analysis

#### Quantitative data

Data from the online questionnaires were downloaded directly into a database and checked for logic errors. Data analysis, using standard statistical packages, will allow us to answer key questions concerning the sexual behaviour of ethnic minority MSM in Britain, HIV prevalence, their use of sexual health services as well as their experience of stigma and discrimination. In all analyses ethnic minority MSM and "key migrant" MSM will be compared with white British men. In addition we will be able to find out what people working in sexual health services (both treatment and prevention) think are the needs of ethnic minority MSM.

Univariate analyses will be conducted using the chi-squared test for categorical data and unpaired t-tests for normally distributed, continuous data. Associations between binary dependent variables and possible predictors will be investigated using logistic regression. Multivariable logistic regression will be used to identify variables that are independently and significantly associated with outcomes of interest. The samples vary in size but in general are sufficiently large to allow us to detect statistically significant differences between and within different groups at the 5% level of significance. For example, a sample size of 13,000 white British MSM and 200 Indian MSM will provide more than 90% power to detect a difference in HIV prevalence between the two groups of 6% at a significance level of 0.05 (i.e. HIV prevalence among white British MSM = 9%, HIV prevalence among Indian MSM = 3%).

#### Qualitative data

We conducted email interviews with 67 ethnic minority MSM. Participants were from Black African, Black Caribbean, Indian, Pakistani, Chinese, Malaysian and mixed backgrounds. Ages ranged from 21 to 44 years (mean = 28). Transcript data will be entered into NVivo, a software program for organizing and conducting text searches. Using content analysis with an iterative process for coding, categories and concepts that emerge from the text will be identified and subsequently linked together. Using a grounded theory approach, all transcripts will be examined by two members of the research team in an effort to identify broad themes that emerge from the data.

Table 4 summarises how many people completed a questionnaire in the different samples between August 2007 and April 2008. Just over half the ethnic minority MSM lived in London compared with less than twenty percent of the white MSM. This reflects national census data that shows that nearly half the ethnic minority population of the UK lives in London [10].

**Table 4:** Number of respondents in the different national samples

	London	Outside London	Total
Ethnic minority MSM	657	584	1241
Key migrant MSM	252	162	416
White British MSM	2602	11115	13717
Sexual health clinic staff	152	199	364*
Sexual health promotion staff	33	91	124

* 13 sexual health clinic staff did not indicate where they worked

In addition 67 ethnic minority MSM were included in the qualitative arm of the study

All analysis of the data from the study will take place at City University London, Department of Public Health and will be undertaken by members of the research team.

## Discussion

Innovative Internet-based research methods have allowed us to examine the sexual health of ethnic minority men who have sex with men (MSM) living in Britain [28]. Using an online questionnaire we were able to survey MSM across Britain from a diverse range of backgrounds. Our sample of 1241 ethnic minority MSM is the largest recruited to date in the UK. In addition, we conducted qualitative interviews online among more than 60 ethnic minority respondents. To the best of our knowledge this is the first study in Britain to have conducted qualitative interviews among MSM in this way. Our study throws into sharp focus the Internet's potential for conducting qualitative and quantitative research among a hard-to-reach group of men [30,31].

A key feature of the study was the involvement of stakeholders. To recruit ethnic minority MSM we worked closely with community groups and non-governmental organizations that work with ethnic minority MSM, as well as with Gaydar, the most popular dating site for MSM in the UK. A close collaboration with the British Association for Sexual Health and HIV (BASHH) allowed us to recruit clinical staff from sexual health clinics in our target towns and cities in Britain. An equally close relationship with the Terrence Higgins Trust CHAPS partnership facilitated the recruitment of sexual health promotion staff from around Britain.

Results from the study will be published on websites likely to be accessed by participants (e.g. http://ukblackout.co.uk, http://gaysia.co.uk) and the findings are likely to receive publicity in the mainstream gay press and on HIV information websites (e.g. Aidsmap http://www.aidsmap.com/). Findings will be published in peer-reviewed journals. Key articles will be sent to service providers who participated in the project. Members of the research team will present findings from the study at relevant national and international conferences such as the International AIDS Society (IAS), British Association of Sexual Health and HIV (BASHH), British HIV Association (BHIVA) and International Society for STD Research (ISSTDR) meetings.

The results of the study will improve our understanding of the sexual health of ethnic minority MSM in Britain and their needs. In addition, they will help in the formulation of innovative interventions among ethnic minority MSM for primary and secondary STI/HIV prevention.

## Ethics approval

The research project received Multisite Research Ethics Committee (MREC) approval from South West MREC in January 2007 (ref: 06/MRE06/71)

## Competing interests

The authors declare that they have no competing interests.

## Authors' contributions

JE, SN, NL and JA conceived the study; JE, EM, SN, NL and JA participated in its design; JE was responsible for overall project management; EM was responsible for both the quantitative and qualitative arms of the study; RD was responsible for quantitative data analysis; JE drafted the manuscript with input from EM and RD. All authors read, revised and approved the final manuscript.

## Appendices

### Appendix 1

#### Research team

City University London

Professor Jonathan Elford, Principal Investigator

Dr Eamonn McKeown, Senior Research Fellow

Dr Rita Doerner, Research Fellow

Terrence Higgins Trust, Bristol

Simon Nelson, Co-investigator

Homerton University Hospital NHS Foundation Trust Hospital, London

Professor Jane Anderson, Co-investigator

University of Bern, Switzerland

Dr Nicola Low, Co-investigator

#### Community representatives

*Lesbian and Gay Coalition Against Racism*   Denis Fernando

*Black Gay Men's Action Group*   Robert Berkeley

*Wisethoughts*   Subodh Rathod

*LYC London*   Chris Stransom, Ian Bowman

*NAZ Project London*   Carlos Corredor

*Imaan*   Yusef Gojikian, Hanaan

#### Advisory group

*African HIV Research Forum*   Dr Ade Fakoye

*British Association for Sexual Health and HIV (BASHH)*   Dr Angela Robinson

*Camden Primary Care Trust (PCT)*   John Zavuga, David Smith

*Gaydar/Qsoft*   Henry Badenhurst

*GMFA*   Carl Burnell

*Greater London Authority*   Dr Cheikh Traore

*NAZ Project London*   Margareth Rungarara, Bryan Texeira

*Sigma Research*   Peter Weatherburn

*Terrence Higgins Trust*   Marc Thompson, Will Nutland

*UK Black Out*   Andrew Prince

### Appendix 2

#### Internet sites and email lists where we advertised the MESH project to reach ethnic minority MSM

*Community websites*

Wisethoughts

UK blackout

BGMag

Outburst UK

Mysalaam

Gaysia

Bengayliz

LYC London

Somali Gay Community

Imaan

*Health promotion websites*

HGL Birmingham

GMFA

LGBT Health Scotland

*Social networking websites*

Gaydar

Manhunt.net

Black Gay Chat

World Gay Men

Fitlads.net

*Other websites*

Gumtree

Facebook

*Email lists*

Gaydar

LYC London

THT

Imaan

### Appendix 3

#### Sexual health promotion/HIV prevention projects in the 15 target towns and cities in Britain

*London*   GMFA, THT London, Camden PCT

*Birmingham*   THT Midlands

*Bradford*   Yorkshire MESMAC

*Brighton*   THT South

*Bristol*   THT West

*Cardiff*   THT Cymru

*Leeds*   Yorkshire MESMAC

*Leicester*   Trade

*Liverpool*   The Armistead Project

*Luton*   Men4Men Sexual Health Outreach Project

*Manchester*   Lesbian and Gay Foundation

*Newcastle*   MESMAC North East

*Nottingham*   Healthy Gay Nottingham

*Sheffield*   Centre for HIV and Sexual Health

*Glasgow*   THT Scotland

## Pre-publication history

The pre-publication history for this paper can be accessed here:

http://www.biomedcentral.com/1471-2458/10/419/prepub

## Supplementary Material

Additional file 1 **Sexual health clinics in the 15 target towns and cities in Britain**. A list of the sexual health clinics in Britain that were (i) invited to participate in the research and (ii) agreed to take part. Click here for file
