# Supplementary material for: Sexual health of ethnic minority MSM in Britain (MESH project): design and methods
Source: BMC Public Health. 2010 Jul 14;10:419. doi: 10.1186/1471-2458-10-419 (PMC2916902; doi:10.1186/1471-2458-10-419)
Supplement: Additional file 1 — Sexual health clinics in the 15 target towns and cities in Britain. A list of the sexual health clinics in Britain that were (i) invited to participate in the research and (ii) agreed to take part. [file 1471-2458-10-419-S1.DOC]

###### Additional file 1. Sexual health clinics in the 15 target towns and cities in Britain

| **City/town** | **NHS Trust** | **Hospital/clinic** | **Agreed to participate** | **R&D approval** |
| --- | --- | --- | --- | --- |
|  |  |  |  |  |
| **London** |  |  |  |  |
|  | Camden PCT | Mortimer Market, UCH | Y | Y |
|  | Royal Free Hampstead NHS Trust | Marlborough Clinic | Y | Y |
|  | Homerton Hospital NHS Trust | Dept of Sexual Health, Homerton Hospital | Y | Y |
|  | Haringay Health Care NHS Trust | St Anne’s Hospital Sexual Health Centre | Y | Y |
|  | North Middlesex Hospital NHS Trust | Coleridge Unit, North Middlesex Hospital | Y | Y |
|  | Newham Community Health Services NHS Trust | Greenway Centre, Newham General Hospital | Y | Y |
|  | The Royal London Hospitals Trust | Bart’s Sexual Health Centre, St Bartholomew’s Hospital | Y | Y |
|  | Barts and the London NHS Trust | Ambrose King Centre, Royal London Hospital | Y | Y |
|  | Camden PCT | Archway Sexual Health Clinic | Y | Y |
|  | North West London Hospitals NHS Trust | Patrick Clements Clinic, Middlesex Hosp | Y | Y |
|  | Chelsea and Westminster Healthcare NHS Trust | West London Centre for Sexual Health | Y | Y |
|  | Chelsea and Westminster Healthcare NHS Trust | John Hunter Clinic | Y | Y |
|  | Chelsea and Westminster Healthcare NHS Trust | Kobler Clinic | Y | Y |
|  | Chelsea and Westminster Healthcare NHS Trust | Victoria Clinic | Y | Y |
|  | St Mary’s NHS Trust | Jefferiss Wing Centre for Sexual Health, St Mary’s Hospital | Y | Y |
|  | Guy’s and St Thomas’ Hospitals NHS Trust | Lydia Department, St Thomas’ Hospital | Y | Y |
|  | Guy’s and St Thomas’ Hospitals NHS Trust | Lloyd Clinic, Guy’s Hospital | Y | Y |
|  | Kings College Hospital NHS Trust | Caldecot Centre, King's College Hospital | Y | Y |

| **City/town** | | **NHS Trust** | **Hospital/clinic** | **Agreed to participate** | **R&D approval** |
| --- | --- | --- | --- | --- | --- |
|  | |  |  |  |  |
| **London, cont** | |  |  |  |  |
|  | Mayday Healthcare NHS Trust | | Department of GU Medicine, Mayday University Hospital |  |  |
|  | Forest Healthcare NHS Trust | | Whipps Cross Hospital Dept. of Sexual Health | Y | Y |
|  | St George’s Healthcare NHS Trust | | Roehampton Clinic | Y | N |
|  | | St George’s Healthcare NHS Trust | Courtyard clinic, St George’s Hospital | Y | N |
|  | | Hillingdon Hospital NHS Trust | Tudor Wing, Hillingdon Hospital | N | - |
|  | | Northwick Park and St Mark’s Hospitals NHS Trust | Northwick Park Hospital | N | - |
|  | | Barnet and Chase Farm Hospitals NHS Trust | Clare Simpson House, Barnet Hospital | N | - |
|  | | Enfield Community Care NHS Trust | The Town Clinic | N | - |
|  | | Barking, Havering and Redbridge Hospitals NHS Trust | The Sydnenham Centre, Barking Hospital | N | - |
|  | | Bromley Hospitals NHS Trust | Dept of GU Medicine, Beckenham Hospital | N | -. |
|  | | Kingston Hospital NHS Trust | Wolverton Centre for Sexual Health | N | - |
|  | | Ealing Hospital NHS Trust | Ealing Hospital Pasteur Suite | N | - |

| **City/town** | | **NHS Trust** | **Hospital/clinic** | | **Agreed to participate** | **R&D approval** |
| --- | --- | --- | --- | --- | --- | --- |
|  | |  |  | |  |  |
| **Outside London** | |  |  | |  |  |
| Birmingham | | Heart of Birmingham Teaching PCT | Whittal Street Clinic | | Y | Y |
|  | | Birmingham and Heartlands NHS Trust | Hawthorn House | | Y | Y |
| Bradford | | Bradford Hospitals NHS Trust | St Luke’s Hospital | | Y | Y |
| Brighton | | Brighton and Sussex University Hospitals Trust | Claude Nicol Centre, The Royal County Hospital | | Y | Y |
| Bristol | | United Bristol Healthcare Trust | Bristol Royal Infirmary Milne Centre | | Y | Y |
| Cardiff | | Cardiff and Vale NHS Trust | Cardiff Royal Infirmary | | Y | Y |
| Leeds | | United Leeds Teaching Hospitals NHS Trust | Sunnybank Wing, The General Infirmary at Leeds | | Y | Y |
| Liverpool | | Royal Liverpool and Broadgreen University Hospital NHS Trust | Royal Liverpool Hospital | | Y | Y |
| Luton | | Luton and Dunstable Hospital NHS Trust | Luton and Dunstable Hospital | | Y | Y |
| Manchester | Central Manchester and Manchester Children’s University Hospitals NHS Trust | | | Manchester Royal Infirmary | Y | Y |
|  | North Manchester Healthcare NHS Trust | | | North Manchester General Hospital | Y | Y |
|  | South Manchester University Hospital NHS Trust | | | Withington Hospital | Y | Y |
|  | Trafford Healthcare NHS Trust | | | Trafford General Hospital | Y | Y |
| Newcastle | Newcastle Primary Care Trust | | | Newcastle General Hospital | Y | Y |
| Nottingham | Nottingham City Hospital NHS Trust | | | Nottingham City Hospital | Y | Y |
| Sheffield | Sheffield Teaching Hospitals Trust | | | Royal Hallamshire Hospital | Y | Y |
| Glasgow | Genitourinary medicine services | | | The Sandyford Initiative | Y | Y |
| Leicester | Leicester Royal Infirmary NHS Trust | | | Leicester Royal Infirmary | Y | Y |
| Salford* | Salford Royal Hospitals NHS Trust | | | Hope Hospital Manchester | N | -. |

Salford is part of the Greater Manchester conurbation
